# How air pollution influences clinical management of respiratory diseases. A case-crossover study in Milan

**DOI:** 10.1186/1465-9921-13-95

**Published:** 2012-10-18

**Authors:** Pierachille Santus, Antonio Russo, Enzo Madonini, Luigi Allegra, Francesco Blasi, Stefano Centanni, Antonio Miadonna, Gianfranco Schiraldi, Sandro Amaducci

**Affiliations:** 1 Dipartimento Cardio-Respiratorio, Unità Operativa di Pneumologia, San Carlo Borromeo Hospital, Via Pio II, 3, 20153, Milan, Italy; 2 Unità di Epidemiologia e Biostatistica, San Carlo Borromeo Hospital, Milan, Italy; 3 Dipartimento Fisiopatologia Medico-Chirurgica, Università degli Studi di Milano, IRCCS Fondazione Ospedale Maggiore Cà Granda, Milan, Italy; 4 Dipartimento di Scienze della Salute, Università degli Studi di Milano, Respiratory Unit, San Paolo Hospital, Milan, Italy; 5 Divisione di Medicina Interna e Pneumologia, Fatebenefratelli Hospital, Milan, Italy; 6 Struttura Complessa di Pneumologia, Niguarda Ca’ Granda Hospital, Milan, Italy; 7 Pneumologia Riabilitativa, Fondazione Salvatore Maugeri, Istituto Scientifico di Riabilitazione di Milano, IRCCS, Milan, Italy

**Keywords:** Air pollution, COPD, Asthma, Emergency admissions, Public health

## Abstract

**Background:**

Environmental pollution is a known risk factor for multiple diseases and furthermore increases rate of hospitalisations. We investigated the correlation between emergency room admissions (ERAs) of the general population for respiratory diseases and the environmental pollutant levels in Milan, a metropolis in northern Italy.

**Methods:**

We collected data from 45770 ERAs for respiratory diseases. A time-stratified case-crossover design was used to investigate the association between air pollution levels and ERAs for acute respiratory conditions. The effects of air pollutants were investigated at lag 0 to lag 5, lag 0–2 and lag 3–5 in both single and multi-pollutant models, adjusted for daily weather variables.

**Results:**

An increase in ozone (O_3_) levels at lag 3–5 was associated with a 78% increase in the number of ERAs for asthma, especially during the warm season. Exposure to carbon monoxide (CO) proved to be a risk factor for pneumonia at lag 0–2 and in the warm season increased the risk of ERA by 66%. A significant association was found between ERAs for COPD exacerbation and levels of sulphur dioxide (SO_2_), CO, nitrate dioxide (NO_2_), and particulate matter (PM_10_ and PM_2.5_). The multipollutant model that includes all pollutants showed a significant association between CO (26%) and ERA for upper respiratory tract diseases at lag 0–2. For chronic obstructive pulmonary disease (COPD) exacerbations, only CO (OR 1.19) showed a significant association.

**Conclusions:**

Exposure to environmental pollution, even at typical low levels, can increase the risk of ERA for acute respiratory diseases and exacerbation of obstructive lung diseases in the general population.

## Background

Today, in industrialized countries as in the low and middle income ones, the association of respiratory diseases and environmental pollution represents a relevant social and health problem. Various studies have been performed to evaluate the effects of air pollution on health status demonstrating that not only West Europe and North America are involved in this problem but also developing countries [1-3]. However, further investigation, in the form of both human and animal studies, as well as in vitro, needs to be performed to fully comprehend the relationship between air pollution and human morbidity and mortality. Several experiences have demonstrated the adverse health effects of environmental pollution [4] particularly concerning respiratory [5] and cardio-vascular diseases [6]. Special attention will be focused on the respiratory system which is the first point of contact with air pollutants in humans. Seaton et al. [7] proposed that inhaled air pollutants induce alveolar inflammation with activation of cellular and molecular chain mechanisms promoting lung disease exacerbations. The level of environmental pollution has a role in human health as well as temporal exposure. The differences in pollutant exposure between single individuals is another interesting point and probably subjects at risk, such as people with asthma and other chronic respiratory diseases, may be more likely affected by particulate pollution if they live or work close to busy roads or other sources of air pollution [8]. Furthermore, some high-risk groups have been identified, such as children, [9] and elderly people [10]. Acute effects studies have found significant correlation between concentrations of particulate matter with an aerodynamic diameter ≤ 10 or 2.5 micron (PM_10_ and PM_2.5_), NO_2_, SO_2_, and disease exacerbations, emergency admissions, hospitalizations and mortality [5]. The main sources of exposure in the general population are combustion processes, traffic, automobile service stations, gasoline transfer, exhaust fumes from motor vehicles, and industrial emissions [11]. The association between acute or prolonged effect of particulate air pollution and emergency room visits or hospital admission for respiratory conditions and asthma represent, even today, an important question. In fact, many previous experiences have reported that pollutants promote health problems in different countries thus including Italian cites as well [12-17]. Indeed, every country and city has their geographical, socio-political and health regulations, so performing large number of studies may offer the possibility to better understand the global problem of air pollution. Thus, the aim of our study was to investigate the correlation between ERA for respiratory diseases in the general population and the environmental pollutant levels in Milan, the largest metropolis of northern Italy.

## Methods

### Study population

This study (named POEMI: *POllution and Emergencies in MIlan*) was carried out in the Emergency Departments (EDs) of the main five city hospitals in Milan, Italy. These five hospitals covered about 80% of the total number of ERAs in Milan. Patients were included in the study carried out between January 1, 2007 and December 31^st^, 2008 if they were upper respiratory tract infections (URTI), acute asthma, pneumonia or exacerbation of chronic obstructive pulmonary disease (COPD). An MD reviewed the ED records and recorded admission date, age, gender, race, postal code, ED diagnosis and outcome (either discharge or death) for each patient.

The study was firstly approved by the central ethics committee and then by the other individual research ethics boards.

### Data on air pollution exposure

Outdoor air pollution levels within the city of Milan are measured by eight air quality monitoring stations managed by the Regional Environmental Protection Agency (ARPA). At the time of this study, the number of city stations for monitoring nitrogen oxides (NO, NO_2_, NO_X_) were eight, for CO five, O_3_ and PM_10_ three, benzene, toluene and xylenes two, and SO_2_ and PM_2.5_ one.

The methods and technologies used to measure air concentrations are designated at national and international level (i.e., UV fluorescence for SO_2_, chemiluminescence for NO_X_, UV photometry for O_3_, IR photometry for CO etc.). As for particulate matter, PM_10_ and PM_2.5_ are currently obtained using beta-ray attenuation monitors and corrected TEOM (Tapered Element Oscillating Microbalance). The ARPA has determined correction factors for particulate matter levels based on gravimetric and TEOM measure comparison. The parallel geometric instruments have been placed in different sites and for different period during the year; the correction factors have been verified retrospectively and subsequently applied.

Five monitoring stations are classified as "traffic" sites (typically CO and benzene stations) and are located mostly along main streets or squares in Milan. Three stations are "urban background" sites, dedicated to the measurement of SO_2_ and PM_2.5_ and are situated in urban areas far from major streets. One of them is located inside a large urban park. PM_10_ is measured at two traffic stations and one urban background station. Nitrogen oxides are measured at all of the stations.

None of the stations is classified as "industrial" because industrial sources are not significant sources of pollution in the city of Milan. Automobiles and domestic heating plants are the main sources of atmospheric emissions in Milan.

Pollutant concentrations measured hourly by the regional monitoring network have been aggregated by day according to the specific air quality standards required by European laws. For example, the average concentration of SO_2_, particulate matter and benzene over 24-hours was measured, whereas the maximum hourly concentrations were considered for O_3_ and NO_2_ etc. Finally, registered daily values of each air pollutant were from all the stations, in order to obtain their average level and to assess the global environmental situation of the city. Temperature and humidity levels were derived from the archive of the regional meteorological service and they refer to data measured at "urban" stations in the city.

To obtain a complete dataset over the study period, a few missing values were estimated with the assumption of a constant (i.e., not time-dependent) proportional relationship between the mean values measured by each monitoring station. The number of missing values is very small. In the study period (2 years) there are no missing observations for NO_2_, CO, O_3_, and PM10. The days with missing values were 34 for SO_2_, 48 for PM_2,5_ and 40 for C_6_H_6_, C_7_H_8_, C_8_H_10_. These data were estimated by ARPA based on the trend of concentrations detected by monitoring stations not belonging to the Municipality of Milan, but as much as possible next to it and positioned in locations similar to the urban environment of Milan.

## Design and statistical analysis

We used a case-crossover design [18] to assess the risk of ERA for acute conditions based on exposure to various pollutants. The case-crossover design controls for long-term trends and seasonal changes. We used the time-stratified approach [19] to select control days. Thus, every seventh day from the event day within the same month and year of the event day was considered a control. This approach desumes seasons by matching months and year. It also partly controls other variables, such as weather, because all comparisons are made within the same month and on the same day of the week. The event day is termed lag 0, and the day before the event day is lag 1. The day before lag 1 is lag 2, and so forth. To assess the excess risk of ERA based on several potential predictors and to control confounding factors, conditional regression models were fitted using odds ratio (OR) and corresponding 95% confidence intervals (95% CI). All models included daily weather variables (including temperature, temperature squared, and humidity on lag 1). To assess pollution exposure, same-day average exposures and lagged intervals extending from 1 to 5 days before the case or control event were obtained. Additionally we averaged levels from lag 0 to lag 2 to explore an early effect. Stratified analyses of exposure based on the average exposure at lag 0 to lag 2 based on age, class, gender, race and season was undertaken to evaluate effect modification. The results of the analyses were expressed as an OR to quantify the increase in risk based on a corresponding increased exposure of 10 μg/m^3^ of NO_2,_ PM_10,_ and PM_2.5_, 5 μg/m^3^ of SO_2_ and 1 μg/m3 of CO. For benzene (C_6_H_6_), toluene (C_7_H_8_), and xylene (C_8_H_10_), an interquartile range was measured based on the daily mean levels of each air pollutant over the entire study period. To explore cumulative exposure we constructed dummy variables that assumes the value of 1 when the levels for each pollutant at lag 3–5 were larger than the value of the interquartile range (Table 1).

**Table 1:** (**A**) **Daily environmental variables**; **and** (**B**) **Spearman correlation coefficients for these variables**

	**Warm season**		**Cold season**		***Total***		**Percentiles**			
	**(April-September)**		**(October-March)**							
**A.**	**Mean**	**SD**	**Mean**	**SD**	**Mean**	**SD**	**25th**	**50th**	**75th**	**IQR**
**C**_**6**_**H**_**6**_	1.41	0.72	3.99	2.06	2.70	2.01	1.18	2.05	3.78	2.60
**C**_**9**_**H**_**6**_**N**_**2**_**O**_**2**_	5.23	3.36	12.51	7.84	8.87	7.04	3.90	6.83	11.86	7.96
**C**_**8**_**H**_**10**_	2.35	1.85	6.44	4.84	4.39	4.19	1.62	3.09	5.86	4.24
**PM**_**10**_	31.08	11.96	63.13	33.17	47.08	29.62	26.92	37.00	60.15	33.23
**PM**_**2**.**5**_	16.20	9.70	49.44	28.35	32.80	26.92	13.00	22.00	47.00	34.00
**SO**_**2**_	3.75	2.69	4.51	4.25	4.13	3.57	1.04	3.61	6.00	4.96
**NO**_**2**_	89.47	28.01	115.79	34.23	102.62	33.91	79.71	99.43	121.71	42.00
**CO**	1.03	0.27	1.95	0.60	1.49	0.65	1.00	1.33	1.86	0.86
**O**_**3**_	109.42	33.69	39.05	24.70	74.29	45.95	32.67	68.50	111.67	79.00
**B.**	**C**_**6**_**H**_**6**_	**C**_**9**_**H**_**6**_**N**_**2**_**O**_**2**_	**C**_**8**_**H**_**10**_	**PM**_**10**_	**PM**_**2**.**5**_	**SO**_**2**_	**NO**_**2**_	**CO**	**O**_**3**_	
**WARM SEASON(April-September)**										
**C**_**6**_**H**_**6**_	1.00	0.88	0.87	0.49	0.43	−0.13	0.61	0.49	0.11	
**C**_**9**_**H**_**6**_**N**_**2**_**O**_**2**_	0.88	1.00	0.84	0.44	0.40	0.01	0.58	0.47	0.06	
**C**_**8**_**H**_**10**_	0.78	0.90	1.00	0.45	0.40	−0.01	0.65	0.57	0.00	
**PM**_**10**_	0.77	0.67	0.58	1.00	0.75	−0.08	0.52	0.52	0.21	
**PM**_**2**.**5**_	0.76	0.63	0.54	0.96	1.00	−0.17	0.51	0.54	−0.01	
**SO**_**2**_	0.51	0.35	0.30	0.46	0.44	1.00	−0.20	−0.20	0.17	
**NO**_**2**_	0.67	0.70	0.64	0.54	0.48	0.30	1.00	0.67	0.12	
**CO**	0.78	0.69	0.61	0.60	0.60	0.34	0.59	1.00	−0.18	
**O**_**3**_	−0.44	−0.27	−0.24	−0.39	−0.46	−0.33	0.07	−0.41	1.00	
**COLD SEASON(October-March)**										

Multi-pollutant models were fitted for each specific disease, using the mean values from lag 0–2 for all statistically significant pollutants from the univariate analyses.

## Results

### Study subjects and pollutants data

During the two-year study period, 45770 ERAs were recorded. The demographic and clinical characteristics of this study population are shown in (Table 2). The number of total ERAs was comparable between the two study years, but higher rates of ERAs were observed during the cold season (61.3% of admissions occurred from October to March).

**Table 2:** Distribution of daily ERAs for acute respiratory diseases according to several characteristics

**Variable**	**Asthma**	**URTI**	**Pneumonia**	**COPD exacerbation**	**Total**
**Age class**					
0-4	821	17971	832	-	**19624**
5-19	601	7660	513	-	**8774**
20-34	718	2901	384	-	**4003**
35-54	899	2552	760	108	**4319**
55-65	172	744	442	185	**1543**
65-74	161	1054	763	437	**2415**
75+	197	1805	1995	1095	**5092**
**Gender**					
Males	1918	18784	3125	1033	**24860**
Females	1651	15903	2564	792	**20910**
**Race**					
Black	312	1401	136	30	**1879**
Asian	134	967	110	4	**1215**
Caucasian	2377	29617	4963	1635	**38592**
Hispanic	222	1570	145	4	**1941**
Unknown	524	1132	335	152	**2143**
**Hospitalization**					
No	2978	31923	1801	499	**37201**
Yes	591	2764	3888	1326	**8569**
**Year**					
2007	1936	17212	2917	960	**23025**
2008	1633	17475	2772	865	**22745**
**Season**					
Warm	2049	12908	2087	666	**17710**
Cool	1520	21779	3602	1159	**28060**
**Total**	**3569**	**34687**	**5689**	**1825**	**45770**

URTI were responsible for the largest number of ERAs (75.7%), mainly due to the high proportion of children up to 19 years of age in this study. Pneumonia, asthma and COPD exacerbation accounted for 12.4%, 7.9% and 4.0% of ERAs, respectively. The 45770 total ERAs resulted in 8569 hospital admissions. Summary statistics for air pollutants and Spearman correlation coefficients are included in Table 1. Daily NO_2_ and SO_2_ levels were higher during the winter season, as opposed to the warmer period of the year, even if the difference between the two was lower than for other pollutants. However, an increase in the daily levels of polycyclic aromatic hydrocarbons (C_6_H_6_, C_7_H_10_, C_8_H_10_), PM_10_, PM_2.5_ and CO occurred in the cold season, and an increase in O_3_ levels occurred in the warm season. There was a high correlation among several pollutants; in particular, benzene, other polycyclic aromatic hydrocarbons and NO_2_ were highly correlated in the warm season, and PM_10_, PM_2.5_, CO and NO_2_ were highly correlated in the cool season and inversely related to O_3_ levels.

### Upper respiratory tract infections

The results of the single-day lags are illustrated in (Table 3). Increases in all the pollutants were significantly correlated with ERAs, with the exception of ozone. Single pollutant models exploring the association between pollution levels at lag 3 to lag 5 and ERAs demonstrate an increase in risk of ERAs from 3% to 10% per one interquartile increase in pollutant concentration (Table 3).

**Table 3:** Association of ERAs and upper respiratory tract infections

**(A)**	**lag 0**	**lag 1**	**lag 2**	**lag 3**	**lag 4**	**lag 5**	**lag 0**-**2**	↑ **3 of 5 days**
*SO*_*2*_ (*5* μ*g*/*m*^*3*^)	1.010 (0.989-1.031)	1.056 (1.035-1.077)	1.072 (1.052-1.093)	1.104 (1.083-1.124)	1.096 (1.076-1.117)	1.076 (1.056-1.096)	1.076 (1.049-1.103)	1.098 (1.061-1.137)
*CO* (*1 mg*/*m*^*3*^)	1.101 (1.073-1.129)	1.144 (1.116-1.172)	1.124 (1.096-1.152)	1.144 (1.115-1.172)	1.143 (1.114-1.172)	1.122 (1.093-1.152)	1.174 (1.140-1.209)	0.987 (0.952-1.023)
*NO*_*2*_ (*10* μ*g*/*m*^*3*^)	0.999 (0.995-1.003)	1.012 (1.008-1.016)	1.013 (1.009-1.016)	1.016 (1.012-1.020)	1.017 (1.013-1.021)	1.009 (1.005-1.013)	1.012 (1.007-1.017)	1.059 (1.029-1.089)
*O*_*3*_ (*10* μ*g*/*m*^*3*^)	0.999 (0.993-1.006)	0.999 (0.992-1.006)	0.992 (0.986-0.999)	0.986 (0.980-0.991)	0.989 (0.983-0.994)	0.983 (0.978-0.989)	0.994 (0.985-1.003)	1.060 (1.009-1.113)
*PM*_*10*_ (*10* μ*g*/*m*^*3*^)	1.019 (1.014-1.024)	1.022 (1.017-1.026)	1.019 (1.014-1.024)	1.019 (1.014-1.024)	1.020 (1.016-1.025)	1.022 (1.017-1.027)	1.029 (1.024-1.035)	1.075 (1.038-1.113)
*PM*_*2*.*5*_ (*10* μ*g*/*m*^*3*^)	1.018 (1.013-1.024)	1.019 (1.013-1.024)	1.016 (1.011-1.022)	1.020 (1.014-1.025)	1.024 (1.019-1.030)	1.027 (1.022-1.033)	1.026 (1.019-1.033)	1.028 (0.995-1.062)
*C*_*6*_*H*_*6*_ (*2*.*6 mg*/*m*^*3*^)	1.020 (1.001-1.041)	1.063 (1.043-1.083)	1.062 (1.042-1.083)	1.074 (1.054-1.095)	1.085 (1.064-1.107)	1.081 (1.060-1.103)	1.076 (1.050-1.102)	1.051 (1.013-1.090)
*C*_*7*_*H*_*8*_ (*7*.*96 mg*/*m*^*3*^)	0.975 (0.958 (0.991)	1.027 (1.011-1.045)	1.025 (1.008-1.042)	1.043 (1.026-1.060)	1.050 (1.033-1.068)	1.036 (1.019-1.054)	1.015 (0.994-1.037)	1.030 (0.993-1.068)
*C*_*8*_*H*_*10*_ (*4*.*24 mg*/*m*^*3*^)	0.983 (0.969 (0.998)	1.018 (1.004-1.033)	1.023 (1.008-1.038)	1.024 (1.009-1.038)	1.023 (1.008-1.038)	1.011 (0.996-1.026)	1.015 (0.995-1.035)	1.005 (0.974-1.037)
**(B)**	**Age group**		**Gender**		**Race**		**Season**	
	**≤16**	**≥65**	**Males**	**Females**	**Caucasian**	**Others**	**Warm**	**Cold**
*SO*_*2*_ (*5* μ*g*/*m*^*3*^)	1.074 (1.044-1.106)	1.085 (0.990-1.189)	1.076 (1.040-1.113)	1.075 (1.036-1.116)	1.067 (1.039-1.097)	1.125 (1.054-1.201)	**0**.**873** (**0**.**815**-**0**.**936**)	**1**.**118** (**1**.**088**-**1**.**149**)
*CO* (*1 mg*/*m*^*3*^)	1.187 (1.148-1.227)	1.112 (0.995-1.243)	1.192 (1.146-1.240)	1.153 (1.104-1.204)	1.179 (1.142-1.216)	1.148 (1.063-1.239)	**0**.**885** (**0**.**785**-**0**.**997**)	**1**.**203** (**1**.**166**-**1**.**241**)
*NO*_*2*_ (*10* μ*g*/*m*^*3*^)	1.016 (1.010-1.022)	0.999 (0.980-1.017)	1.011 (1.004-1.018)	1.013 (1.005-1.020)	1.013 (1.008-1.019)	1.004 (0.991-1.017)	**0**.**983** (**0**.**972**-**0**.**995**)	**1**.**020** (**1**.**014**-**1**.**025**)
*O*_*3*_ (*10* μ*g*/*m*^*3*^)	0.989 (0.979-1.000)	0.998 (0.967-1.029)	0.994 (0.982-1.007)	0.993 (0.980-1.007)	0.997 (0.987-1.006)	0.978 (0.955-1.002)	**1**.**022** (**1**.**009**-**1**.**035**)	**0**.**965** (**0**.**952** (**0**.**977**)
*PM*_*10*_ (*10* μ*g*/*m*^*3*^)	1.031 (1.024-1.038)	1.013 (0.992-1.035)	1.029 (1.021-1.037)	1.030 (1.022-1.039)	1.027 (1.021-1.034)	1.041 (1.025-1.058)	1.028 (0.999-1.058)	1.029 (1.023-1.035)
*PM*_*2*.*5*_ (*10* μ*g*/*m*^*3*^)	1.028 (1.020-1.036)	1.004 (0.980-1.028)	1.028 (1.018-1.037)	1.024 (1.015-1.034)	1.025 (1.018-1.032)	1.030 (1.012-1.048)	0.997 (0.965-1.030)	1.026 (1.020-1.033)
*C*_*6*_*H*_*6*_ (*2*.*6 mg*/*m*^*3*^)	1.094 (1.064-1.124)	1.016 (0.928-1.113)	1.079 (1.045-1.115)	1.072 (1.035-1.111)	1.073 (1.045-1.101)	1.091 (1.025-1.162)	1.099 (0.979-1.234)	1.077 (1.050-1.104)
*C*_*7*_*H*_*8*_ (*7*.*96 mg*/*m*^*3*^)	1.030 (1.005-1.056)	0.953 (0.880-1.033)	1.013 (0.984-1.043)	1.018 (0.986-1.050)	1.019 (0.996-1.043)	0.986 (0.932-1.044)	0.904 (0.840-0.973)	1.028 (1.005-1.052)
*C*_*8*_*H*_*10*_ (*4*.*24 mg*/*m*^*3*^)	1.022 (1.000-1.045)	0.976 (0.908-1.050)	1.014 (0.987-1.041)	1.015 (0.986-1.045)	1.016 (0.995-1.038)	0.998 (0.947-1.051)	0.880 (0.810-0.955)	1.024 (1.003-1.045)

**KEY**: Results are expressed as Odds Ratio (OR) and corresponding 95% confidence intervals (95% CI) with (A) various pollutants at various lags and (B) in various subgroups at lag 0–2 from conditional logistic regression analysis including terms for daily weather variables (1-day lag for temperature, humidity and temperature squared).

The effects of the pollutants in the subgroups were similar. However, a seasonal effect emerged. Increases in ozone correlated with increases in URTI in the warm season. On the other hand, PM_10_ and PM_2.5_ were only associated with increased risk for ERA for URTI in the cold season. In a multipollutant model using the lag 0–2 structure for all pollutants associated with URTI in the single pollutant model, only CO (OR 1.261; 95% CI 1.199-1.327) and PM_10_ (OR 1.02; 95% CI 1.014-1.032) retained statistically significant association with URTI ERAs.

### Asthma

Adjusted odd ratios for ERAs for asthma due to increases in pollution are displayed in (Table 4). Increases in ozone levels from lag 1 to lag 5 corresponded to increases in ERAs for asthma.

**Table 4:** Association of ERAs and asthma

**(A)**	**lag 0**	**lag 1**	**lag 2**	**lag 3**	**lag 4**	**lag 5**	**lag 0**-**2**	↑ **3 of 5 days**
*SO*_*2*_ (*5* μ*g*/*m*^*3*^)	0.975 (0.904-1.052)	0.976 (0.906-1.053)	1.059 (0.986-1.137)	1.041 (0.970-1.117)	1.050 (0.980-1.125)	1.021 (0.952-1.094)	1.008 (0.919-1.106)	0.970 (0.852-1.104)
*CO* (*1 mg*/*m*^*3*^)	1.032 (0.938-1.134)	1.030 (0.938-1.132)	1.003 (0.912-1.104)	1.041 (0.945-1.147)	1.114 (1.011-1.226)	1.142 (1.037-1.257)	1.031 (0.921-1.154)	1.063 (0.932-1.211)
*NO*_*2*_ (*10* μ*g*/*m*^*3*^)	0.968 (0.954-0.981)	0.972 (0.958-0.986)	0.997 (0.983-1.011)	1.013 (0.999-1.027)	1.034 (1.021-1.048)	1.023 (1.009-1.037)	0.967 (0.951-0.985)	0.980 (0.896-1.071)
*O*_*3*_ (*10* μ*g*/*m*^*3*^)	1.010 (0.991-1.029)	1.052 (1.029-1.076)	1.052 (1.033-1.072)	1.061 (1.043-1.078)	1.044 (1.028-1.060)	1.039 (1.024-1.055)	1.068 (1.040-1.097)	1.781 (1.584-2.002)
*PM*_*10*_ (*10* μ*g*/*m*^*3*^)	0.998 (0.981-1.016)	1.005 (0.987-1.024)	1.014 (0.996-1.033)	1.020 (1.002-1.038)	1.015 (0.997-1.032)	1.014 (0.996-1.031)	1.009 (0.987-1.031)	1.044 (0.919-1.186)
*PM*_*2*.*5*_ (*10* μ*g*/*m*^*3*^)	0.993 (0.973-1.014)	0.991 (0.970-1.011)	1.000 (0.980-1.021)	1.003 (0.983-1.023)	1.001 (0.981-1.021)	1.011 (0.991-1.031)	0.992 (0.967-1.017)	1.076 (0.954-1.213)
*C*_*6*_*H*_*6*_ (*2*.*6 mg*/*m*^*3*^)	0.941 (0.873-1.014)	0.920 (0.854-0.992)	0.949 (0.881-1.023)	0.980 (0.910-1.056)	1.034 (0.957-1.117)	1.025 (0.949-1.108)	0.905 (0.825-0.992)	0.955 (0.835-1.093))
*C*_*7*_*H*_*8*_ (*7*.*96 mg*/*m*^*3*^)	0.947 (0.891-1.006)	0.947 (0.890-1.006)	0.965 (0.908-1.026)	0.981 (0.923-1.044)	1.036 (0.974-1.102)	1.023 (0.961-1.090)	0.924 (0.855 (0.999	1.044 (0.912-1.196)
*C*_*8*_*H*_*10*_ (*4*.*24 mg*/*m*^*3*^)	0.926 (0.878 (0.977)	0.934 (0.886 (0.986)	0.953 (0.902-1.005)	0.983 (0.932-1.037)	1.016 (0.964-1.071)	1.001 (0.950-1.055)	0.894 (0.833 (0.959	0.942 (0.850-1.043)
**(B)**	**Age group**		**Gender**		**Race**		**Season**	
	**≤16**	**≥65**	**Males**	**Females**	**Caucasian**	**Others**	**Warm**	**Cold**
*SO*_*2*_ (*5* μ*g*/*m*^*3*^)	0.975 (0.850-1.117)	1.099 (0.846-1.428)	0.901 (0.792-1.025)	1.135 (0.993-1.297)	1.020 (0.917-1.133)	0.964 (0.795-1.170)	0.986 (0.815-1.194)	1.028 (0.923-1.144)
*CO* (*1 mg*/*m*^*3*^)	1.059 (0.901-1.243)	1.472 (1.048- 2.067)	1.082 (0.921-1.271)	0.985 (0.841-1.155)	1.127 (0.993-1.278)	0.724 (0.559- 0.937)	1.179 (0.869-1.599)	1.011 (0.890-1.148)
*NO*_*2*_ (*10* μ*g*/*m*^*3*^)	1.001 (0.975-1.027)	1.008 (0.957-1.063)	0.980 (0.957-1.004)	0.954 (0.930 -0.978)	0.993 (0.973-1.014)	0.910 (0.881-0.940)	0.943 (0.918-0.970)	0.993 (0.970-1.016)
*O*_*3*_ (*10* μ*g*/*m*^*3*^)	1.021 (0.977-1.067)	1.034 (0.944-1.133)	1.062 (1.024-1.100)	1.077 (1.034-1.121)	1.022 (0.989-1.056)	**1**.**162** (**1**.**110**-**1**.**217**)	**1**.**104** (**1**.**068**-**1**.**142**)	1.005 (0.959-1.053)
*PM*_*10*_ (*10* μ*g*/*m*^*3*^)	0.996 (0.964-1.029)	1.030 (0.964-1.101)	0.999 (0.969-1.031)	1.019 (0.988-1.051)	1.027 (1.003-1.053)	0.933 (0.887-0.983)	0.959 (0.893-1.030)	1.009 (0.985-1.033)
*PM*_*2*.*5*_ (*10* μ*g*/*m*^*3*^)	0.983 (0.948-1.020)	1.033 (0.956-1.117)	0.983 (0.949-1.018)	1.002 (0.967-1.038)	1.021 (0.993-1.050)	0.882 (0.833-0.935)	0.846 (0.783- 0.913)	1.006 (0.979-1.033)
*C*_*6*_*H*_*6*_ (*2*.*6 mg*/*m*^*3*^)	0.948 (0.831-1.081)	1.098 (0.832-1.447)	0.931 (0.816-1.061)	0.884 (0.775-1.007)	1.035 (0.936-1.145)	0.504 (0.401-0.633)	0.462 (0.344- 0.619)	1.005 (0.911-1.108)
*C*_*7*_*H*_*8*_ (*7*.*96 mg*/*m*^*3*^)	0.975 (0.870-1.092)	1.241 (0.994-1.549)	0.952 (0.854-1.062)	0.897 (0.802-1.004)	0.976 (0.894-1.066)	0.777 (0.654-0.925)	0.813 (0.679- 0.973)	0.979 (0.895-1.071)
*C*_*8*_*H*_*10*_ (*4*.*24 mg*/*m*^*3*^)	0.973 (0.881-1.074)	1.128 (0.914-1.393)	0.904 (0.818- 0.998)	0.885 (0.801- 0.978)	0.982 (0.909-1.061)	0.618 (0.523-0.731)	0.591 (0.491-0.712)	0.982 (0.907-1.062)

**KEY**: Results are expressed as Odds Ratio (OR) and corresponding 95% confidence intervals (95% CI) with (A) various pollutants at various lags and (B) in various subgroups at lag 0–2 from conditional logistic regression analysis including terms for daily weather variables (1-day lag for temperature, humidity and temperature squared). Models for asthma included terms for total pollen levels as tertiles.

The effect was strongest among non-Caucasian subjects (OR 1.162; 95% CI 1.110-1.217) and during the warm season (OR 1.104; 95% CI 1.068-1.142). CO appeared to be higher during the warm season and increased risk of asthma attacks with ERA (47%) in the elderly population. Almost no significant gender difference was found between asthma and ERAs. Only PM_10_ could influence asthma attacks in Caucasian subjects, however with a very little risk (OR 1.027; 95% CI 1.003-1.053).

### Pneumonia

Table 5 shows the case-crossover analysis results for pneumonia ERAs. Reported data underline an acute effect of CO, SO_2_ and PM_10_ on ERAs for pneumonia particularly evident at lag 0 and 1. Moreover, CO increases risk of ERA at lag 0–2, highlighting also a cumulative effect (OR 1.108; 95% CI 1.028-1.194). Stratified analysis for lag 0–2 days showed a 66% risk increase for ERAs for pneumonia in the warm season. A significant ERA increase can be found in the Caucasian female population with over 65 years of age. The young female population seems to have ERAs for pneumonia during the cold season with a risk of 16% every 5 μg/m^3^ increase of SO_2_. PM_10_ and PM_2.5_ could represent a risk factor for pneumonia ERA in warm season with an increased risk of respectively 8 and 10%.

**Table 5:** Association of ERAs and pneumonia

**(A)**	**lag 0**	**lag 1**	**lag 2**	**lag 3**	**lag 4**	**lag 5**	**lag 0**-**2**	↑ **3 of 5 days**
*SO*_*2*_ (*5* μ*g*/*m*^*3*^)	1.010 (0.960-1.062)	1.056 (1.004-1.110)	1.041 (0.991-1.094)	1.012 (0.965-1.061)	0.998 (0.952-1.046)	1.018 (0.971-1.067)	1.058 (0.993-1.128)	1.043 (0.955-1.139)
*CO* (*1 mg*/*m*^*3*^)	1.108 (1.039-1.180)	1.065 (1.000-1.134)	1.055 (0.992-1.122)	1.064 (0.999-1.132)	1.047 (0.982-1.117)	1.047 (0.983-1.115)	1.108 (1.028-1.194)	0.947 (0.869-1.033)
*NO*_*2*_ (*10* μ*g*/*m*^*3*^)	1.009 (0.999-1.019)	1.002 (0.992-1.013)	1.002 (0.992-1.013)	1.007 (0.998-1.017)	1.002 (0.992-1.012)	0.999 (0.989-1.009)	1.007 (0.994-1.020)	1.007 (0.940-1.079)
*O*_*3*_ (*10* μ*g*/*m*^*3*^)	1.003 (0.988-1.019)	0.999 (0.980-1.017)	0.992 (0.977-1.008)	0.993 (0.979-1.007)	0.985 (0.972-0.999)	0.984 (0.970-0.997)	0.997 (0.974-1.019)	0.910 (0.805-1.029)
*PM*_*10*_ (*10* μ*g*/*m*^*3*^)	1.013 (1.001-1.025)	1.002 (0.990-1.014)	1.009 (0.997-1.021)	1.015 (1.003-1.027)	1.009 (0.997-1.020)	1.013 (1.001-1.024)	1.011 (0.997-1.026)	1.071 (0.985-1.165)
*PM*_*2*.*5*_ (*10* μ*g*/*m*^*3*^)	1.007 (0.994-1.021)	1.000 (0.987-1.014)	1.006 (0.993-1.020)	1.011 (0.997-1.024)	1.010 (0.997-1.023)	1.014 (1.001-1.027)	1.007 (0.990-1.023)	1.009 (0.932-1.093)
*C*_*6*_*H*_*6*_ (*2*.*6 mg*/*m*^*3*^)	1.023 (0.973-1.074)	0.975 (0.927-1.025)	1.005 (0.957-1.056)	1.017 (0.969-1.067)	0.994 (0.945-1.045)	1.027 (0.977-1.078)	1.001 (0.941-1.065)	0.953 (0.873-1.041)
*C*_*7*_*H*_*8*_ (*7*.*96 mg*/*m*^*3*^)	1.018 (0.976-1.062)	0.980 (0.939-1.023)	0.998 (0.957-1.040)	1.009 (0.968-1.052)	0.988 (0.947-1.031)	0.990 (0.950-1.033)	0.998 (0.945-1.054)	0.964 (0.884-1.052)
*C*_*8*_*H*_*10*_ (*4*.*24 mg*/*m*^*3*^)	1.017 (0.980-1.056)	0.971 (0.935-1.009)	0.989 (0.953-1.026)	0.987 (0.951-1.024)	0.967 (0.932-1.003)	0.989 (0.953-1.026)	0.987 (0.939-1.038)	1.003 (0.929-1.082)
**(B)**	**Age group**		**Gender**		**Race**		**Season**	
	**≤16**	**≥65**	**Males**	**Females**	**Caucasian**	**Others**	**Warm**	**Cold**
*SO*_*2*_ (*5* μ*g*/*m*^*3*^)	1.165 (1.033-1.314)	1.043 (0.947-1.149)	1.004 (0.920-1.096)	1.126 (1.025-1.237)	1.058 (0.989-1.133)	1.059 (0.880-1.274)	0.994 (0.834-1.186)	1.072 (1.000-1.148)
*CO* (*1 mg*/*m*^*3*^)	1.094 (0.947-1.263)	1.188 (1.063-1.328)	1.092 (0.985-1.210)	1.129 (1.012-1.260)	1.102 (1.016-1.194)	1.132 (0.925-1.385)	**1**.**657** (**1**.**248**- **2**.**199**)	1.061 (0.979-1.149)
*NO*_*2*_ (*10* μ*g*/*m*^*3*^)	0.998 (0.973-1.024)	1.019 (1.000-1.038)	1.001 (0.984-1.019)	1.014 (0.995-1.033)	1.010 (0.996-1.024)	0.985 (0.950-1.021)	1.020 (0.992-1.049)	1.000 (0.985-1.015)
*O*_*3*_ (*10* μ*g*/*m*^*3*^)	0.967 (0.919-1.016)	0.983 (0.952-1.014)	0.980 (0.950-1.010)	1.017 (0.984-1.050)	0.982 (0.958-1.006)	1.104 (1.035-1.177)	1.005 (0.974-1.038)	0.986 (0.955-1.018)
*PM*_*10*_ (*10* μ*g*/*m*^*3*^)	1.016 (0.988-1.045)	1.017 (0.995-1.039)	1.012 (0.993-1.032)	1.011 (0.989-1.033)	1.011 (0.996-1.027)	1.009 (0.966-1.054)	1.081 (1.008-1.159)	1.007 (0.992-1.022)
*PM*_*2*.*5*_ (*10* μ*g*/*m*^*3*^)	1.008 (0.977-1.041)	1.019 (0.995-1.043)	1.010 (0.988-1.033)	1.002 (0.978-1.027)	1.008 (0.991-1.026)	0.988 (0.942-1.037)	1.101 (1.018-1.190)	1.002 (0.985-1.019)
*C6H6* (*2*.*6 mg*/*m*^*3*^)	1.019 (0.906-1.146)	1.046 (0.954-1.147)	0.957 (0.879-1.043)	1.053 (0.963-1.153)	1.021 (0.955-1.090)	0.857 (0.718-1.024)	1.095 (0.828-1.448)	0.989 (0.928-1.055)
*C*_*7*_*H*_*8*_ (*7*.*96 mg*/*m*^*3*^)	0.981 (0.882-1.091)	1.038 (0.959-1.124)	0.967 (0.897-1.042)	1.035 (0.955-1.121)	0.994 (0.937-1.053)	0.991 (0.849-1.157)	1.055 (0.898-1.239)	0.978 (0.922-1.038)
*C*_*8*_*H*_*10*_ (*4*.*24 mg*/*m*^*3*^)	0.964 (0.877-1.060)	1.019 (0.947-1.097)	0.964 (0.900-1.033)	1.013 (0.942-1.090)	0.990 (0.938-1.045)	0.928 (0.806-1.068)	1.204 (0.994-1.459)	0.959 (0.909-1.012)

**KEY**: Results are expressed as Odds Ratio (OR) and corresponding 95% confidence intervals (95% CI) with (A) various pollutants at various lags and (B) in various subgroups at lag 0–2 from conditional logistic regression analysis including terms for daily weather variables (1-day lag for temperature, humidity and temperature squared).

### COPD exacerbation

A significant association was found between ERAs for COPD exacerbation and increases in SO_2_, CO and PM_10_ on previous days when measured as linear effect (Table 6). Analysis of lag 0–2 shows an increase in ERA for COPD exacerbation of 3%, 14% and 24% for increases in PM_10_, SO_2_, and CO, respectively. An increase of one interquartile of PM_10_ for 3 days in a period of 5 days prior to the event produced an increased risk of ERA of 21%. There was a 3-fold increased risk of ERA (OR 3.124; 95% CI 1.845-5.289) among non-White populations with corresponding increases in CO.

**Table 6:** Association of ERAs and COPD exacerbation

**(A)**	**lag 0**	**lag 1**	**lag 2**	**lag 3**	**lag 4**	**lag 5**	**lag 0**-**2**	↑ **3 of 5 days**
*SO*_*2*_ (*5* μ*g*/*m*^*3*^)	1.035 (0.951-1.127)	1.105 (1.015-1.203)	1.121 (1.031-1.218)	1.116 (1.028-1.210)	1.058 (0.973-1.150)	1.085 (1.002-1.176)	1.144 (1.027-1.274)	1.092 (0.936-1.274)
*CO* (*1 mg*/*m*^*3*^)	1.145 (1.019-1.286)	1.229 (1.100-1.373)	1.114 (0.994-1.249)	1.087 (0.969-1.219)	1.106 (0.984-1.243)	1.049 (0.937-1.174)	1.236 (1.080-1.415)	1.108 (0.952-1.289)
*NO*_*2*_ (*10* μ*g*/*m*^*3*^)	1.008 (0.989-1.026)	1.033 (1.015-1.053)	1.014 (0.996-1.033)	1.016 (0.998-1.034)	1.005 (0.988-1.023)	1.004 (0.987-1.021)	1.029 (1.005-1.052)	1.157 (1.023-1.309)
*O*_*3*_ (*10* μ*g*/*m*^*3*^)	0.987 (0.961-1.013)	0.957 (0.926-0.989)	0.960 (0.933-0.987)	0.969 (0.945-0.993)	0.969 (0.947-0.993)	0.985 (0.963-1.008)	0.944 (0.908-0.982)	0.864 (0.701-1.065)
*PM*_*10*_ (*10* μ*g*/*m*^*3*^)	1.022 (1.001-1.044	1.028 (1.006-1.049)	1.023 (1.001-1.045)	1.013 (0.993-1.035)	1.017 (0.997-1.038)	1.017 (0.997-1.037)	1.036 (1.010-1.063)	1.213 (1.046-1.407)
*PM*_*2*.*5*_ (*10* μ*g*/*m*^*3*^)	1.017 (0.992-1.042)	1.023 (0.999-1.048)	1.023 (0.999-1.048)	1.015 (0.992-1.039)	1.023 (1.000-1.046)	1.020 (0.998-1.043)	1.031 (1.001-1.062)	1.096 (0.950-1.265)
*C*_*6*_*H*_*6*_ (*2*.*6 mg*/*m*^*3*^)	1.015 (0.926-1.111)	1.082 (0.991-1.182)	1.047 (0.957-1.145)	1.008 (0.923-1.100)	0.989 (0.903-1.084)	0.994 (0.911-1.084)	1.076 (0.962-1.203)	1.096 (0.937-1.283)
*C*_*7*_*H*_*8*_ (*7*.*96 mg*/*m*^*3*^)	0.997 (0.924-1.076)	1.082 (1.004-1.166)	1.031 (0.955-1.113)	1.021 (0.948-1.099)	0.970 (0.899-1.046)	0.961 (0.894-1.034)	1.061 (0.963-1.170)	0.935 (0.800-1.092)
*C*_*8*_*H*_*10*_ (*4*.*24 mg*/*m*^*3*^)	1.046 (0.981-1.116)	1.083 (1.016-1.155)	1.051 (0.986-1.120)	1.029 (0.967-1.095)	0.988 (0.928-1.051)	0.948 (0.892-1.009)	1.112 (1.019-1.212)	0.995 (0.868-1.141)
**(B)**	**Age group**		**Gender**		**Race**		**Season**	
	**≤16**	**≥65**	**Males**	**Females**	**Caucasian**	**Others**	**Warm**	**Cold**
*SO*_*2*_ (*5* μ*g*/*m*^*3*^)		1.095 (0.975-1.231)	1.193 (1.036-1.375)	1.077 (0.911-1.273)	1.139 (1.017-1.274)	1.227 (0.828-1.817)	0.905 (0.678-1.209)	1.205 (1.071-1.356)
*CO* (*1 mg*/*m*^*3*^)		1.205 (1.041-1.395)	1.346 (1.128-1.607)	1.092 (0.886-1.348)	**1**.**155** (**1**.**003**-**1**.**330**)	**3**.**124** (**1**.**845**-**5**.**289**)	1.745 (1.024 2.975)	1.144 (0.990-1.323)
*NO*_*2*_ (*10* μ*g*/*m*^*3*^)		1.028 (1.003-1.054)	1.035 (1.005-1.066)	1.019 (0.983-1.056)	1.022 (0.998-1.047)	1.147 (1.048-1.256)	1.070 (1.016-1.126)	1.012 (0.985-1.039)
*O*_*3*_ (*10* μ*g*/*m*^*3*^)		0.945 (0.905- 0.986)	0.892 (0.845-0.941)	1.009 (0.952-1.069)	0.956 (0.918- 0.997)	0.820 (0.714 -0.942)	0.973 (0.921-1.028)	0.916 (0.864 (0.970)
*PM*_*10*_ (*10* μ*g*/*m*^*3*^)		1.029 (1.000-1.058)	1.049 (1.015-1.085)	1.017 (0.977-1.059)	1.036 (1.008-1.064)	1.127 (1.017-1.248)	1.133 (0.991-1.295)	1.027 (1.000-1.054)
*PM*_*2*.*5*_ (*10* μ*g*/*m*^*3*^)		1.025 (0.992-1.058)	1.043 (1.004-1.083)	1.013 (0.967-1.061)	1.034 (1.002-1.066)	1.115 (0.995-1.251)	1.108 (0.950-1.293)	1.024 (0.993-1.055)
*C*_*6*_*H*_*6*_ (*2*.*6 mg*/*m*^*3*^)		1.070 (0.948-1.208)	1.120 (0.968-1.297)	1.016 (0.855-1.208)	1.078 (0.960-1.210)	1.443 (0.914 2.276)	0.991 (0.587-1.675)	1.065 (0.949-1.195)
*C*_*7*_*H*_*8*_ (*7*.*96 mg*/*m*^*3*^)		1.054 (0.948-1.172)	1.111 (0.978-1.263)	0.994 (0.855-1.156)	1.051 (0.949-1.163)	1.478 (0.998 2.188)	0.886 (0.628-1.250)	1.054 (0.950-1.169)
*C*_*8*_*H*_*10*_ (*4*.*24 mg*/*m*^*3*^)		1.069 (0.972-1.177)	1.166 (1.041-1.306)	1.037 (0.907-1.186)	1.041 (0.947-1.144)	1.908 (1.421 2.563)	0.959 (0.664-1.384)	1.085 (0.989-1.191)

**KEY**: Results are expressed as Odds Ratio (OR) and corresponding 95% confidence intervals (95% CI) with (A) various pollutants at various lags and (B) in various subgroups at lag 0–2 from conditional logistic regression analysis including terms for daily weather variables (1-day lag for temperature, humidity and temperature squared).

The multipollutant model of CO, NO_2_ and PM_10_ using lag 0–2 average values showed a significant effect of CO increases on ERAs for COPD with an OR of 1.193 (95% CI 1.035-1.375).

## Discussion

Our study prospectively analysed the impact of air pollution on ERAs for lung disease among the general population of Milan. The results identified a positive association between ERAs for respiratory diseases and ambient exposure to pollutants such as PM_10_, O_3_, CO and SO_2_.

### Climatic considerations

Recently, concern on the effects of meteorological factors, such as weather and temperature, on population health has increased. Some studies [11,16] have shown that mortality rate from respiratory diseases has a direct correlation to air temperature increase, even if the mortality-temperature relationship has generally a U-shape, pointing out the issue of the extremes of temperature. For this reason, we reported the seasonal variation of pollutants during the cold and the warm periods. As reported in the literature, O_3_ is one of the most important atmospheric gases involved in photochemical reactions [17]. O_3_ levels were higher in the warm months in our study, a finding that is in line with scientific evidence that this atmospheric gas is usually dominant during the summer season [20]. On the other side, the most important source of CO in the Mediterranean areas is the incomplete combustion from cars and trucks, especially from gasoline engines operating at low speeds and during winter [21]. This data has been confirmed in our study too, as CO levels increased in the winter months (Table 1). As is already well known, atmospheric chemistry transport model simulations suggest that summer time O_3_ levels are greatly enhanced throughout the entire Mediterranean troposphere, contributing substantially to the radiative forcing of climate [22]. As reported in Table 1, there was a high correlation among several pollutants; in particular, benzene, other polycyclic aromatic hydrocarbons and NO_2_ were highly correlated in the warm season. O_3_ in the troposphere, is a product of photochemical reactions that involve primary pollutants, such as oxides of nitrogen (NOx), CO and volatile organic compounds from industrial and traffic emission [20]. Hence, the O_3_ high level and reactivity, justified besides its presence also the increase of other pollutants. The PM_10_, PM_2.5_, CO and NO_2_ correlation underline an environmental situation characteristically present during the cool season and justified the inverse correlation between these pollutants and O_3_ levels.

### Clinical implications

It is also important to consider the impact of environmental air on health. Increases in ERAs due to URTI have certain socioeconomic repercussions. In fact, our data from single-pollutant models showed that increases of CO, SO_2_, PM_10_ and PM_2.5_ increase URTI ERA by 17%, 7%, 3% and 2.6%, respectively. This finding is in line with a previous paper published by Jaakkola et al. who found a significant association between upper respiratory infections and living in an air-polluted area [23]. In our study, there were nearly 23000 subjects ≤16 years of age who presented with URTIs, and approximately 20% of these subjects were non-EU members. The high number of URTIs among children was the same finding of the previous study performed in Southwest Milan where CO and NO_2_ played a principle role during acute pollutant exposure [14]. The high number of these patients has socioeconomic repercussions on the health system (e.g. higher costs in terms of human and instrumental resources) and on the management of emergency departments.

### COPD and asthma: environmental and biological considerations

Unlike URTIs, COPD and asthma can have serious complications, so more detailed considerations should be made. Approximately 25% of COPD exacerbations are likely due to non-infectious causes [24]. Air pollution may be one of the main non-infectious triggers of COPD exacerbations. Anderson et al. demonstrated in a previous study that air pollution was associated to daily hospital admissions for COPD with an increase of 50 μg/m^3^ in daily mean level of pollutant (SO_2_, black smoke and total suspended particulates) [12]. Interesting at this point, is the fact that the mean increase values of pollutant we considered were different from that used by APHEA Project and appear lower (Table 6). In particular, our study showed that ERAs for COPD exacerbation significantly increased with an acute exposure at lag 0–2, to CO (23%), SO_2_ (14%), PM_10_ (3.6%) and PM_2.5_ (3%). Only PM_10_ displayed a significant association at lag 3–5 (OR 1.21), indicating that the effect of PM_10_ is linked to cumulative and acute exposure. Of the considered pollutants, the effect of CO on COPD exacerbations should be carefully noted. COPD is more common in older men [25]. In fact, our data demonstrated that for a 1 mg/m^3^ increase of CO there was a 20% increase in ERAs for COPD exacerbations in patients over 65. Patients older than 75 had the highest number of ERAs and 72% (1326) of them were ultimately hospitalised. There were differences in the effects of CO on ERAs by race. However, for PM_10_ exposure there was an increased risk of ERAs (OR 1.12) in non-White subjects, which could be due to poor living conditions or to higher outdoor exposure during the cold seasons. Moreover, our data show that CO levels appear to play a major role in COPD exacerbations during the warm season. This finding follows an acute exposition pattern: in the presence of a 1 mg/m^3^ increase of CO there was a significant increase (OR 1.74) in ERAs for COPD exacerbations. This is likely the result of high outdoor exposure during the spring and summer, as people are more likely to walk outside. However, in our study considering the multipollutant model (Table 1), CO was an environmental agent with increased levels throughout the year. As the CO represents one of the major components of diesel exhaust, coming from vehicular traffic, the chance that subjects could be exposed to this pollutant are high. Subsequently, CO triggers the start of the complex proinflammatory cascade in the airways [26]. The latter observation has been demonstrated in a recent study too, where the authors reported that an exposure to major urban streets increases the cell oxidative stress [27]. Lung inflammation in COPD is also related to lipid cell membrane peroxidation [28,29], so the previous evidence appears in line with our data and may explain the possible increase in COPD bronchial inflammation during exposure to pollutants. Moreover, interesting too SO_2_ appears to play a role in increasing ERAs for COPD exacerbations (OR 1.20). The toxic effects of SO_2_ on the respiratory tract have been demonstrated in experimental studies [30]. It is likely that SO_2_, PM_10_, cold air and a variety of hydrated metal ions contribute to airways cytotoxicity, inflammation and mucociliary clearance dysfunction [31,32]. This process could be responsible for the 1159 ERAs that occurred during the cold season, which represent 63% of all COPD ERAs in our study.

Asthma is an inflammatory disease sensible to a number of substances and environmental factors [33]. Increased bronchial reactivity to O_3_ may contribute to inflammation or airway injury [34]. In more recent studies, high ozone levels were associated with ERAs for asthma during the warm season [35] a finding that has been confirmed by our data. In fact, we also observed the association of ERAs for asthma with a 5 μg/m^3^ increase in O_3_ in any lag, though the findings were more striking with a prolonged exposure (OR 1.78). Our result goes with that by Colais P. et al. and confirms an important role of ozone in inducing an effect on asthmatics airways with a deleterious short impact of air pollution on respiratory morbidity in Italian cities [13]. The stronger associations shown between O_3_ levels and ERAs during the warm season among men and women are consistent with previous findings [35]. The rate of ERAs for asthma in the general population rises by 42% during the cold season and this increase can be attributed to viral infections or to an interaction of infectious and environmental factors. We can presume that during the warm season there are fewer competing causes of asthma exacerbation. Indeed, even the triggers for these apparent seasonal differences are not clear. We can speculate that, younger patients, who represented approximately 60% of our asthma admissions, are more responsive to air pollutants when they occur simultaneously with hot temperatures, high humidity and other meteorological factors. Additionally, the personal pollutant exposure could increase in the spring and summer due to the desire to remain outside for longer periods.

### Pneumonia: public health and biological considerations

Finally, we analysed the rates of ERAs for pneumonia, an important cause of hospitalisation, morbidity and mortality among adults over 65 years of age [36]. Our data suggest that the main pollutants significantly associated with ERA for pneumonia were CO, SO_2_, PM_2.5_ and PM_10_. During the cold season only sulphur dioxide could be related to increased admissions for pneumonia, and these data appear to be in line with that of Martins et al. [37]. This data appears to be contradictory, but can be explained with the tendency of the population to stay in their homes during cold season thus so reducing outdoor exposition. As the SO_2_ showed to be a highly reactive pollutant [30], probably even a short exposure may be able to lead a lung immunosuppressive pathway with a consequent increase of airway infections. PM_2.5_ is a traffic pollutant that has been examined as a risk factor for pneumonia, and our data agree with a study by Zanobetti et al. [38], which demonstrated an association between PM_2.5_ and hospital admissions for pneumonia in an elderly population. Our study population was not limited to older adults, who made up (only) the 48% of the total number of pneumonia ERAs, while the remaining population was represented by subjects less than 65 years old. Moreover, 68% (3888) of those with pneumonia ERAs required hospitalisation and 71% of them were over 65. These data emphasize what has been reported in literature [36,38], that the elderly may be more susceptible to pollution. Paradoxically, the OR is more impressive for pneumonia ERAs during the warm season, a finding that has been observed in previous studies [38]. These pollutants probably priming a cellular and molecular activation of airways with consequent proinflammatory and immuno-suppressant activity [30]. Furthermore, the elderly are more likely to walk outside in warm weather where they are exposed to pollutants. Pollutants act by causing bronchoalveolar irritation and inflammation [39] a common pathway in all (of) the respiratory diseases.

## Conclusions

Our study confirms the association between exposure to pollutants and ERAs for respiratory diseases in a large northern Italian town, based on over two years of systematic data collection. We found health effects in both the young and the elderly, even at the low levels normally found in the general environment. Our data demonstrated that in multipollutant models combined exposure to CO emissions and PM_10_ level were particularly notable, but when the single pollutant models are considered other environmental agents gain relevance. So, Milan environmental management should take all this data into account. The consequences of exposure to pollutants are relevant from both a medical and a social perspective, also in view of the direct costs. We must consider the direct costs of providing treatment for these diseases and the indirect costs due to lost productivity.

## Abbreviations

ERA: Emergency room admission; COPD: Chronic obstructive pulmonary disease; O_3_: Ozone; CO: Carbon monoxide; SO_2_: Sulphur dioxide; NO_2_: Nitrate dioxide; NO_X_: Nitrogen oxides; PM: Particulate metter; ED: Emergency department; URTI: Upper respiratory tract Infection; ARPA: Regional environmental protection agency; UV: Ultra Violet; IR: Infra-red; TEOM: Tapered element oscillating microbalance; OR: Odds ratio; CI: Confidence interval; C_6_H_6_: Benzene; C_7_H_8_: Toluene; C_8_H_10_: Xylene.

## Competing interests

The authors declare that they have no competing interests.

## Authors’ contributions

PS has made study design, data analysis, literature search, data interpretation, writing manuscript. EM has made study design, data analysis, literature search, data interpretation, correcting manuscript. AR has made study design, data analysis, statistical analysis, literature search, data interpretation, writing manuscript. LA has made study design, correcting manuscript. FB has made study design, data analysis, data interpretation, correcting manuscript. SC has made study design, data analysis, data interpretation, correcting manuscript. AM has made study design, correcting manuscript. GS has made study design, correcting manuscript. SA has made study design, data analysis, literature search, data interpretation, correcting manuscript. All authors read and approved the final manuscript.
